# Impaired Cardiovascular Response to Exercise in Patients with Severe Asthma: A Case-Control Study

**DOI:** 10.2174/18743064-v16-e2201170

**Published:** 2022-03-15

**Authors:** Athina Georgopoulou, Laskarina Fotiadou, Stavros Tryfon, Zoi Daniil, Afroditi K. Boutou

**Affiliations:** 1 Department of Respiratory Medicine, G. Papanikolaou Hospital, Thessaloniki, Greece; 2 Postgraduate Course, Medical School and Department of Physical Education, University of Thessaly, Larissa, Greece; 3 Department of Respiratory Medicine, Medical School, University of Thessaly, Larissa, Greece

**Keywords:** Asthma, Physical activity, Exercise capacity, Cardiovascular reserve, Double product, Lung function

## Abstract

**Background:**

Although asthmatics may present reduced exercise capacity, data on their cardiovascular responses during exercise testing have been scarcely investigated. The aim of this pilot case-control study is to test: a) whether double product (DP), an index of cardiovascular reserve, differs among patients with severe and mild-moderate asthma, and b) whether DP is associated with asthma control level, physical activity (PA) and exercise capacity, in asthmatics population.

**Materials and Methods:**

A group of patients with severe asthma (group S) and a matched group of patients with mild-moderate asthma (group M) was studied. All participants completed asthma control and physical activity (IPAC) questionnaires, lung function measurements and six-minute walk test. The exercise capacity (as 6-minute walk distance (6MWD) and corresponding work), the Borg Dyspnea, the rating of perceived excursion and the average PA METS were recorded.

**Results:**

A total of 18 patients were studied. DP at exercise end was significantly lower in group S, compared to group M (16412.2±4732.1 *vs.* 18594.8±3984.4 mmHgXbpm; p=0.041) and was moderately associated with % predicted 6MWD (r=0.592; p=0.001). Group S patients were also presented with lower moderate intensity PA, compared to group M, while exercise capacity was similar between the groups. Asthma control level had no impact on exercise capacity nor PA parameters.

**Conclusion:**

Patients with severe asthma may have impaired cardiovascular reserve as established by DP, even when exercise capacity is indifferent from patients with milder disease. As an easy-to-assess parameter, DP may offer further information in the functional evaluation of these patients.

## 1 INTRODUCTION

Asthma is a heterogeneous chronic disorder characterized by airway inflammation and variable expiratory airflow limitation, with increasing prevalence and substantial burden of morbidity worldwide [1, 2]. Characteristic symptoms include wheezing, shortness of breath, chest tightness and cough, which vary over time in intensity and frequency of occurrence. Intense physical activity may provoke asthma-related symptoms, especially among individuals with insufficient asthma control; thus asthmatic patients often avoid exercise and adopt a sedentary lifestyle [2]. However, recent advances in asthma management have changed this perception, so that the Global Initiative for Asthma (GINA) recommends that asthmatics should be engaged in regular physical activity, as this is proven to improve their overall health status, quality of life, severity of symptoms and even the level of asthma control [1, 3].

The 6-minute walking test (6MWT) is a simple, easy to perform, well-tolerated and widely utilized test for evaluating physical capacity among patients with chronic respiratory or other disorders [4, 5]. The six-minute walk distance (6MWD) is the primary outcome of the 6MWT and a prognostic measure in various diseases [6]. However, during the 6MWT several other parameters can be evaluated, which may offer further clinical information but are far less frequently recorded. The double product, that is the product of peak heart rate and maximum systolic blood pressure, is an index of cardiovascular reserve and an estimate of the maximal performance of the left ventricle, which can be evaluated at the end of 6MWT [7]. However, it has been hardly ever measured in patients with respiratory disorders, while data on its association with disease severity and levels of physical capacity are currently scarce.

Based on the aforementioned, the current study aims to investigate, for the first time: a) whether DP assessed at the end of 6MWT differs between patients with severe and mild/moderate asthma, and b) whether levels of asthma control, physical activity and exercise capacity are associated with exercise DP in asthmatic patients.

## 2 MATERIALS AND METHODS

### 2.1 Population

A group of patients with severe asthma (Group S) and a gender-, age- and body mass index-matched group of asthmatic patients with mild-moderate asthma (Group M), diagnosed according to GINA guidelines (1) who attended the Asthma Clinic of the NHS Department of Respiratory Medicine, G Papanikolaou Hospital between September 2019 and December 2019 constituted the study population. Mild asthma was defined as asthma that was well controlled with Step 1 or Step 2 treatment, that is, with as needed inhaled corticosteroid (ICS)/formoterol alone, or with low intensity maintenance controller treatment. Moderate asthma was defined as asthma that was well controlled with Step 3 treatment, *e.g*., low dose ICS / long-acting beta agonist (LABA) and severe asthma was defined as asthma that required Step 4 or Step 5 treatment (*e.g*., high dose ICS /LABA) to prevent it from becoming “uncontrolled, or asthma that remained “uncontrolled”, despite this treatment [1]. All patients were stable without any exacerbations or hospitalizations due to asthma or other causes or changes in asthma-specific medication in the last three months prior to study entry. Patients with a) uncontrolled asthma, b) serious musculoskeletal disorders that interfered with normal physical activity, c) mental disorders that did not allow comprehending and consenting to the protocol, d) arterial oxygen saturation (SpO_2_) at rest <92%, e) other concomitant respiratory disorders, f) smoking history (current smokers or ex-smokers with smoking history ≥10 pack/years or smoking cessation within less than 12 months) were excluded from study entry. All patients provided informed, written consent for study participation, the principles of the Declaration of Helsinki (http://www.wma.net/en/30publications/10policies /b3/ index.html) were followed, and the Scientific Review Board Committee of General Hospital “G. Papanikolaou” approved the study protocol (138/2019).

In more detail, the patient matching was conducted as follows: All asthmatic patients aged 18-80 years old with asthma diagnosis of at least one year, that had participated in clinical trials regarding asthma in the previous 3 years, and were, so, recorded in a clinical database of the Department for Respiratory Medicine, were theoretically eligible for study recruitment. The database included 42 patients and selection was done according to the following procedure: patients were first separated according to severity. For every patient with severe asthma, we tried to match another patient with mild-moderate asthma and similar BMI, gender and age. Thus, following a procedure of 1:1 matching, we created two groups of 10 patients each. These patients were called and asked to participate in the study and given a new informed consent. However, 2 female patients in group M decided to withdraw their consent, so a group of 8 mild-moderate and a group of 10 severe asthma patients were eventually included in the study protocol.

### 2.2 Study Protocol

This is a pilot case-control study. All study data were prospectively collected. Study patients arrived at the clinic in the morning after receiving their regular medication and undergoing a detailed clinical evaluation. To continue with further testing, patients had to have a priori heart rate <100 beats/min and systolic blood pressure <180 mmHg at the day of evaluation, at rest, or else they were asked to repeat the visit within 3-5 days. All participants then completed:

(a) The International Physical Activity Questionnaire (IPAQ, short form): it is a 9-item, self-completed instrument used for the evaluation of PA, by summing up vigorous, moderate and walking PAs over the previous seven-day period and generating a total physical activity score (PAscore), expressed in MET-minutes per week (MET.min/wk) [8]. A Greek version of IPAQ-short has been previously used in the Eurobarometer survey conducted by the European Union [9].

(b) The Asthma Control Test (ACT): It is a 5-item, self-completed tool with 4-week recall (on symptoms and daily functioning) for identifying those with poorly controlled asthma [10]. Each item is scored with a 5-point scale (1=not controlled at all; 5=totally controlled) and the higher the scores, the greater the asthma control. The scores range from 5 (poor control of asthma) to 25 (complete control of asthma); an ACT score >19 indicates well-controlled asthma. ACT has been translated and validated for the Greek population [11].

(c) The Asthma Control Questionnaire (ACQ): It is a self-completed tool with a 7-day recall of symptoms; the 5-item questionnaire was used in this study (ACQ-5). Each item is scored on a 7-point scale (0=no impairment; 6=maximum impairment) and scores range between 0 (totally controlled) and 6 (severely uncontrolled) [12]. Based on the average values, asthma is well controlled when ACQ <0.75. The ACQ has been previously translated and validated for the Greek population [13].

Following, participants underwent lung function measurement (Omnia Pulmonary Function Testing Lab by COSMED^@,^ Rome, Italy), according to American Thoracic Society (ATS)/ European Respiratory Society (ERS) guidelines [14] including post-bronchodilation Forced Expiratory Volume in 1 second (FEV_1_), Forced Vital Capacity (FVC), Total Lung Capacity (TLC), Residual Volume (RV), Inspiratory Capacity (IC), Peak Expiratory Flow (PEF), Forced Expiratory Flow 25-75 (FEF_25-75_) and lung diffusion (Diffusion Capacity of lung for carbon monoxide; DLCO and carbon monoxide transfer coefficient; KCO). The reference values applied were Global Lung Function Initiative (GLI) 2012 [15]. After at least 30 minutes rest, all patients completed a 6-minute walking test (6MWT) on a flat 33m corridor, followed by 3 minutes of recovery, according to ATS guidelines [16]; the distance walked (6MWD, m) was recorded. SpO_2_, *vs* pulse oxymetry, and systolic and diastolic blood pressure, *vs* cuff manometer, were measured at rest, at the end of exercise test and at the end of recovery. Heart rate was measured at rest, at the end of the test and at 1, 2 and 3 minutes of recovery. The 0-10 Borg Dyspnea (BorgD) [17] and the 0-10 Borg Rating of Perceived Excursion (RPE) [18] were assessed before and at the end of the 6MWT. Double product was calculated as the product of systolic blood pressure and heart rate before 6MWT (rest), at the end of the exercise test and at the end of recovery (3^rd^ minute).

### 2.3 Statistical Analysis

The entire analysis was conducted using the IBM SPSS Statistics 20 (CA, USA). Continuous variables were expressed as mean ± standard deviation (mean ± SD) or median [interquartile range] depending on the normality of distribution, while categorical variables as % percentages. The predicted 6MWD was also calculated based on the equation: predicted 6MWD= 1.250 X height – 2.816 X age -39.07 X gender + 518.853 (where female=1 and male=0) [19]. The %predicted 6MWD was then calculated as (6MWD X 100)/predicted 6MWD. The 6-minute walk distance work (6MWork) was calculated as the product of 6MWD X Bodyweight (m X kg) [20]. The Shapiro-Wilk test was applied to exam the normality of distribution for quantitative variables. The Student’s t-test for Independent Samples or the Mann -Whitney U test was used for group comparisons of continuous variables, depending on their distribution of values and the Chi Square was applied for group comparisons of categorical variables. The Pearson r or the Spearman rho were utilized to indicate correlations between continuous variables. P <0.05 was considered significant for all comparisons.

## 3 RESULTS

### 3.1 Population

Eighteen subjects (14 male, 4 female; age: 53.9±14.9 years) were included in the study. Ten subjects (age range 39-72 years old) presented with severe asthma (Group S) and the rest 8 gender- age- and BMI- matched patients (age range 40-78 years old) presented with mild-moderate asthma (Group M). The baseline characteristics of the study population are presented in Table **1**. Patients’ regular treatment were as follow: ICS/LABA (33.3%), ICS/LABA + long-acting muscarinic agonist (LAMA) (33.3%), ICS/LABA + monoclonal antibody (16.7%), ICS/LABA + leukotriene antagonist (11.1%) and leukotriene antagonist (5.6%). No patient received extra short-acting beta agonist (SABA) before visiting the clinic.

### 3.2 Level of Asthma Control

Based on ACT, 61.1% of patients had good and 38.9% had poor asthma control. Of those with good asthma control, 63.6% had mild-moderate, while 36.4% had severe asthma, while among those with poor asthma control, 14.3% had mild-moderate, while 85.7% had severe asthma (p=0.04). Based on ACQ, 44.4% had good, 27.8% had poor asthma control, while another 27.8% were in an intermediate condition. Among patients with mild-moderate asthma most had well-controlled asthma (87.5% based on ACT and 62.5% based on ACQ) and normal spirometry (75%). In the S group, 40% according to ACT or 30% according to ACQ had controlled asthma, while no patient had normal spirometry (that is normal FEV_1_, normal FVC and normal FEV_1_/FVC).

### 3.3 Level of Physical Activity

Average METs/week were 2916.9±2737.7 and only 4 subjects (22.4%) were complied to physical activity levels, as suggested by WHO for subjects >18 years old. Only three subjects presented high PA, 12 (66.7%) moderate PA and the rest 16.7% PA. Exercise capacity, Borg dyspnea, RPE score and double product are presented in Table **2**.

### 3.4 Group Differences

Double-product at exercise end was significantly lower in Group S, compared to Group M (18594.8±3984.4 *vs.* 16412.2±4732.1 mmHgXbpm; p=0.041), while BorgD was significantly higher (Table **3**). Although total METs were similar between the groups, group S had significantly lower moderate intensity physical activity, compared to Group M. However, 6MWD (both absolute and %predicted), 6MWWork, ACT score, ACQ and RPE were similar between the groups (Table **2**). Moreover, the values of HR during the 1^st^, 2^nd^ and 3^rd^ minutes of recovery between the groups were also similar. Table **3** presents the absolute and the %predicted values of HR at the end of 6MWT for each patient. In M group the HR% predicted was 62.7±11.3%, while in the S group it was 66.8±10.9% (p>0.05), indicating similar exercise intensity between the groups.

When subjects were compared according to asthma control based on ACT, none of the above parameters were found to be different (data not shown).

### 3.5 Correlations

**Fig. (1):**
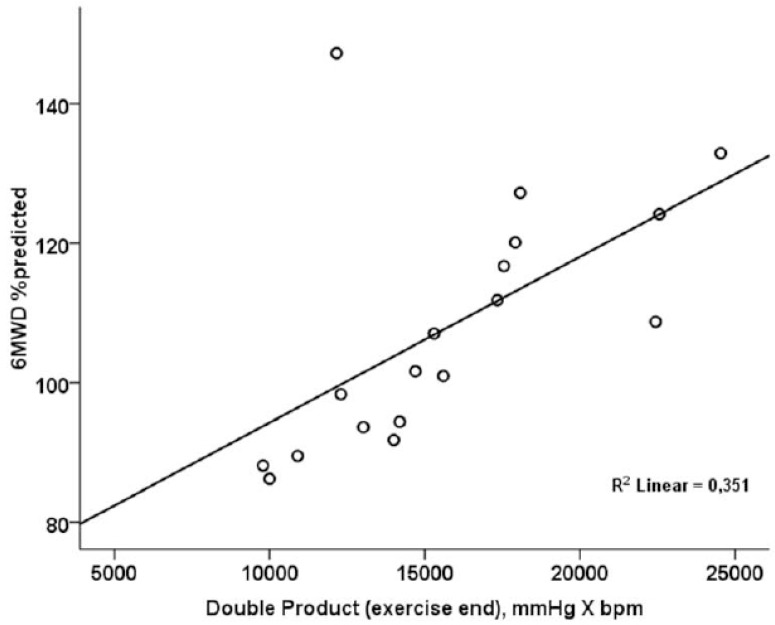
The positive, linear association between double product and 6-minute walking distance (% predicted).

Double product at the end of 6MWT was significantly and positively correlated to 6MWD %predicted (r=0.592; p=0.010) (Fig. **1**).

## 4 DISCUSSION

In this preliminary case-control report, we indicated that patients with severe asthma presented with lower double product at the end of 6-minute walking test, compared to those with mild-moderate asthma. Interestingly, exercise capacity (assessed by both 6MWD and 6MWWork) and overall physical activity (assessed by IPAQ), were similar between the groups. To the author’s knowledge, this is the first study indicating that cardiovascular reserve may be decreased in subjects with severe disease, without evident impairment of exercise capacity.

Both the variables that compose a double product, systolic blood pressure and heart rate, are predictors of disability in the general population [21, 22]. The double product, as an index of myocardial oxygen consumption [23, 24], has been previously used in combination with exercise tests, mostly among patients with cardiovascular disorders. Patients with coronary heart disease presented an attenuated increase in DP during exercise testing [25-27], while DP was significantly increased after 6MWT among patients with heart failure, compared to controls [28]. Data regarding patients with respiratory disorders are currently scarce. Tzani *et al*. indicated that Chronic Obstructive Pulmonary Disease (COPD) patients with dynamic hyperinflation have a poor cardiovascular response to exercise, compared to patients without dynamic hyperinflation, as indicated by lower DP [29]. Reduced left ventricle stroke volume, as a result of a decreased venous return due to high intrathoracic pressure [29], has been proposed as the main reason; however, in our study, resting lung volumes were similar between the groups, while no correlations were established between DP and lung function variables. Deconditioning is another parameter that has been previously associated with changes in DP among healthy subjects [30]. Reduced physical performance, stemming from a sedentary lifestyle, is frequently encountered among asthmatics and has been associated with worse asthma symptoms [31]. In our study, patients with severe asthma presented with significantly lower METs regarding moderate intensity physical activity, confirming these results. Moreover, DP was positively correlated to 6MWD (% predicted), indicating that the higher the exercise capacity, the better the cardiovascular reserve. Since no previous study has investigated the potential role of DP changes in assessing functional capacity and development of dynamic hyperinflation, especially in patients with severe asthma, this should be further studied in the future.

Although exercise capacity assessed by 6-minute walking distance was similar between the groups, certain differences were noted in physical activity intensity, indicating that these two assessments cannot be used indiscriminately in asthmatics. In the study of Janssen *et al*. where exercise capacity and physical activity were separately assessed, these two measures were concomitantly decreased only in one-third of asthmatics; in most of the rest, there was a significant deviation between exercise tolerance as assessed by 6-minute walking distance (“can do”) and daily step count, as assessed by an accelerometer (“do do”) [32]. Moreover, in the study of Verlaet *et al*., less than half of asthmatic male and female patients reached physical activity recommendations [33], confirming our results. Surprisingly, although the level of control has been previously associated with both exercise capacity and physical activity levels [32, 33], in our study, only asthma severity established such an association. Further studies should investigate whether severe asthma is associated with specific permanent changes (respiratory and/or peripheral) that may impact physical capacity, irrespectively of the level of control.

The current report has certain strengths and limitations. The overall number of patients is quite small, and this may be the reason why no group differences were noted in FVC and FEV_1_ values. Moreover, no minimum clinical important difference is reported for DP in literature, while no study power calculation was conducted; however, no outlier was noted regarding DP, and its measured values had a similar trend, so it is highly unlikely results regarding DP to have been different with a bigger sample size. Currently, the majority of published studies refer either to patients with cardiovascular disorders, or to healthy population; the only study that has been conducted in patients with a respiratory disorder, and can be compared to our study, is the one of Tzani *et al*. [29], which included 48 COPD patients. Although these patients were much older with much more severe obstruction, compared to our study, the DP variation within the groups was similar to DP variation in our study. A strength of this pilot study is that the two groups were matched for sex, age and BMI and all data were prospectively collected, minimizing recall bias. Physical activity was not measured using a physical activity monitor, such as an accelerometer, but it was estimated *vs* IPAQ; however, this tool has been repeatedly used for the assessment of physical activity among subjects with chronic respiratory disorders [34, 35].

## CONCLUSION

In conclusion, these preliminary results indicate that patients with severe asthma may have impaired cardiovascular reserve as established by DP at the end of 6-minute walking test, for the first time. As DP is an easy to calculate index, its routine assessment in patients with respiratory disorders may provide further information regarding their functional capacity. Future studies are necessary to investigate the association between DP and maximum exercise capacity, as assessed by cardiopulmonary exercise testing and to reveal the potential impact of asthma severity and level of control on its values.

## Figures and Tables

**Table 1:** Baseline characteristics of the study population.

**Variable**	**Group S**	**Group M**	**p**
Age, y	56.6±10.5	51.38±18.3	NS
Gender, male/female (n)	2/10	2/8	NS
Body Mass Index, kg/m_2_	32.2 (9)	31.6 (8)	NS
ACT	18 (10)	24 (10)	NS
ACQ	0.9 (2.20)	0.5 (2.8)	NS
FEV1, %	73.4 ± 25.6	95.5 ± 12.5	0.004
FVC, %	82.6 ± 15.7	96.3 ± 9.6	0.006
Tiffenau	73.1 ± 11.1	80.5 ± 7.2	NS
PEF,(L)	5.8 ±1.9	6.9 ± 2.6	NS
DLCO, %	78.7 ± 13.4	80.5 ± 10,2	NS
KCO, %	101.5 ± 14.2	95.5 ± 10.1	NS
TLC, %	95.1 ± 17,9	105 ± 6.5	NS
FRC, %	89.8 ± 21.9	100.2 ± 15.5	NS
RV/TLC	113.6 ± 21.9	91.5 ± 32	NS
RV, %	107.4 ± 37.8	93.6 ± 30	NS

ACT: Asthma control test; ACQ: asthma control questionnaire; FEV_1_: Forced Expiratory Volume in 1 second; FVC: Forced Vital Capacity; PEF: Peak Expiratory Flow; DLCO: Diffusion capacity for carbon monoxide; KCO: transfer coefficient; TLC: Total Lung Capacity; FRC: Functional Residual Capacity; RV: Residual Capacity.

**Table 2:** Group differences in physical activity, exercise capacity parameters, and double product.

**Variable**	**Group S**	**Group M**	**p**
PA average, METS	2916.9±2737.7	3104.8±1268.2	NS
Mild PA, METS	414.8±426.2	699.2±943.7	NS
Moderate PA, METS	1770.5±1026.5	2070.9±1494	0.043
Intense PA, METS	720±2113.6	540±703.9	NS
6MWD, m	476.5±62.1	530.6±81.8	NS
6MWD, %	104.3(44.8)	104.9(61)	NS
6MWork, m X kg	41142±6832.2	43865.6±19363.2	NS
BorgD, rest	2 (4)	0 (5)	NS
BorgD, end	3 (8)	0.5 (5)	0.026
RPE, rest	0 (5)	0 (3)	NS
RPE, rest	1 (7)	1 (3)	NS
HR, rest	67.2±8.1	67.9±8.9	NS
HR, end	107.9±14.5	106.8±20.6	NS
SBP, rest	128.5±25.8	127.1±11.6	NS
SBP, end	147.1±32.6	143.8±27.2	NS
DBP, rest	81.5±7.4	76.9±13.9	NS
DBP, end	78±7.9	78.8±16.4	NS
Double Product rest, mmHgXbpm	8598.5±1006.5	8623.1±2178.6	NS
Double Product end, mmHgXbpm	16412±4732.1	18594±3984.4	0.041
Double Product recovery, mmHgXbpm	9488±2155.1	9830±2298.5	NS
HR, 1^st^ minute of recovery	94.1±13.8	87.9±11.9	NS
HR, 2^nd^ minute of recovery	79.9±11.9	80.3±12.2	NS
HR, 3^rd^ minute of recovery	75.9±10.7	75.4±10.1	NS

PA: Physical activity; 6MWD: 6-minute walk distance; 6MWork: 6 minute work; BorgD: Borg Dyspnea; RPE: rate of perceived excursion; HR: Heart rate; SBP: Systolic Blood Pressure; DBP: Diastolic Blood Pressure.

**Table 3:** Absolute HR at the end of 6MWT and %predicted values for each of the patients.

**Patient**	**Absolute HR at 6MWT End**	**% Predicted HR at 6MWT End**	**Group**
1	117	76	S
2	109	62	S
3	102	61	S
4	89	57	S
5	105	61	S
6	141	89	S
7	100	55	S
8	112	75	S
9	111	72	S
10	93	58	S
11	82	46	M
12	129	66	M
13	113	74	M
14	102	63	M
15	120	68	M
16	76	54	M
17	132	79	M
18	100	53	M

HR: Heart rate; 6MWT: 6 minute walking test

## Data Availability

Not applicable.
